# Can We Do Better for Older Adult Research Participants? Clinical Trial Improvements Prompted by a Global Pandemic

**DOI:** 10.1093/geroni/igab046.394

**Published:** 2021-12-17

**Authors:** Susy Stark

**Affiliations:** Washington Unversity, St Louis, Missouri, United States

## Abstract

Retaining older adults in clinical trials has often been a challenge for researchers. Clinical trial procedures, aimed at improving fidelity, often offer barriers to frail older adults who have challenges traveling to medical centers and enduring long clinical assessment visits. During the COVID-19 pandemic, we modified the procedures of two randomized controlled trials. COMPASS: A novel transition program to reduce disability after stroke is a clinical trial examining the efficacy of a transition home program that provides home modifications and self-management strategies compared to stroke education. HARP: Removing home hazards for older adults living in affordable housing is a pragmatic trial examining the effectiveness of a home hazard removal program for residents of low-income housing. Modifications to the trials were designed to reduce human contact but in some cases reduced the burden on trial participants. Modified procedures addressed retention, assessment of endpoints and intervention methods.

